# Full-thickness Macular Hole Formation and Spontaneous Closure Following Vitrectomy Surgery: A Case Report and Review of the Literature

**DOI:** 10.18502/jovr.v21.18238

**Published:** 2026-06-09

**Authors:** Alyssa C. Bonnell, Marcus A. Toral, Jonathan B. Le, Kasra A. Rezaei

**Affiliations:** Department of Ophthalmology, University of Washington, Seattle, WA, USA

**Keywords:** Macular Hole, Spontaneous Closure, Vitreomacular Traction, Vitrectomy

## Abstract

**Purpose:**

To report two cases of macular holes (MHs) that spontaneously formed and closed following pars plana vitrectomy (PPV) and summarize the literature.

**Case Report:**

A 72-year-old man and a 67-year-old woman underwent PPV for repair of rhegmatogenous retinal detachment. Both patients developed MHs following PPV. In both cases, the holes closed without further surgical intervention. A retrospective chart review and literature review were conducted to supplement this study.

**Conclusion:**

Spontaneous MH closure is possible in post-vitrectomized eyes. Small- or medium-sized MHs ( < 400 µm) may be more likely to close spontaneously. Large MHs ( > 400 µm) that form in the setting of vitreomacular traction (VMT) from residual posterior vitreous cortex remnants were found to close after spontaneous VMT release, often within the first month following surgery. Overall, among eyes with spontaneous closure, about two-thirds of MHs in the literature closed spontaneously within the first month following diagnosis, and 4 in 5 holes closed within 3 months. Over one-third of MHs that close spontaneously reopen later, some requiring additional surgery to achieve closure. Sustained closure was seen in this report up to 7 years following closure.

##  INTRODUCTION

Our current understanding of macular hole (MH) development and treatment dates to the 1980s, when Johnson and Gass first proposed that vitreomacular traction (VMT) may lead to MH formation based on clinical observation.^[1]^ These observations prompted the investigation of vitrectomy surgery as a potential treatment for patients with MHs by resolving this traction.^[2]^ While surgery was successful in many cases, by the late 1980s, reports began to emerge of MHs that formed spontaneously following vitrectomy surgery in the absence of obvious traction at the macula.^[3]^ Over the past several decades, there have been further reports of MHs forming after pars plana vitrectomy (PPV) for various indications. The estimated incidence of MH formation following PPV is < 1%.^[4,5]^

Several surgical interventions have been reported to be successful for MHs that form following PPV. However, surgery may not always be necessary, as spontaneous and sustained closure of these holes may be possible. There are a few reports in the literature of spontaneous closure of these MHs.^[5]^ The purpose of this study is to report two cases of full-thickness MH formation and spontaneous closure following vitrectomy surgery. In addition, we present a review of all cases in the literature, summarize the clinical characteristics of eyes that developed MHs and subsequently underwent spontaneous closure following PPV, and discuss the potential clinical implications of these observations. To our knowledge, this is the largest and most comprehensive literature review on this topic to date.

### Case 1

A 72-year-old man with a past ocular history of high myopia, pseudoexfoliation glaucoma (prescribed timolol once per day in the morning and latanoprost nightly in both eyes), steroid-related ocular hypertension, and pseudophakia initially presented with a chief complaint of 2 days of flashing lights in the left eye. On initial evaluation, the patient had a best-corrected visual acuity of 20/25 in both eyes. Intraocular pressures were 15 mmHg and 14 mmHg in the right and left eyes, respectively. Pupillary examination was without a relative afferent pupillary defect in either eye. On dilated fundoscopic examination of the left eye, the patient was found to have a superior macula-sparing rhegmatogenous retinal detachment (RRD) with subretinal fluid spanning from approximately 11 to 1 o'clock with a retinal break at 12 o'clock. He was subsequently taken to the operating room within 3 hours following diagnosis for uncomplicated retinal detachment repair, undergoing 25-gauge PPV, fluid–air exchange, endolaser, and 14% octafluoropropane (C3F8) in his left eye.

**Figure 1:**
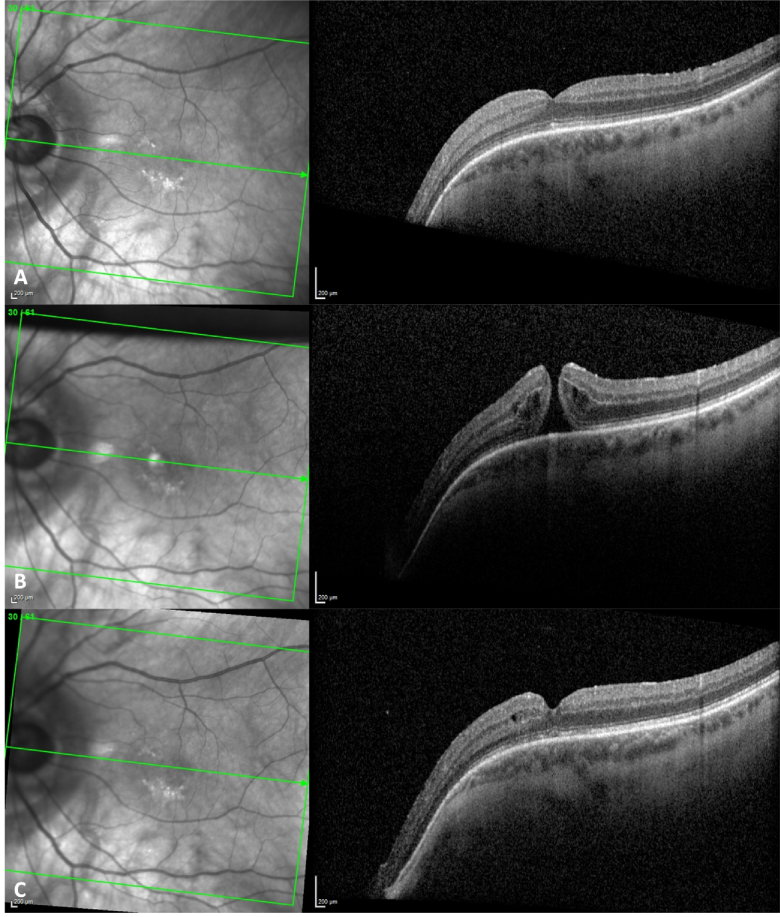
Optical coherence tomography (OCT) demonstrating normal macula architecture following vitrectomy for patient 1 (A). At postoperative month 1, the patient was found to have a small, full-thickness macular hole (MH) (B). By postoperative month 2, the MH was found to have closed and has remained closed through the most recent follow-up, postoperative month 6.

The patient was seen in the clinic postoperatively with a good result [Figure 1A]. However, 4 months postoperatively, the patient was found to have two small macula-sparing RRDs in the left eye without proliferative vitreoretinopathy, located supertemporal and nasal. The patient was subsequently taken back to the operating room within 24 hours following diagnosis and underwent scleral buckling, 25-gauge PPV, fluid–air exchange, endolaser, and 15% C3F8 in the left eye. Postoperatively, the patient was again found to have a good result. OCT imaging was not obtained at postoperative week 1. However, fundoscopic evaluation at this visit revealed a normal macula without MH. At postoperative month 1, the patient's visual acuity was 20/60, and he was found to have a small, full-thickness MH (measuring 61 µm minimum aperture diameter) with trace cystoid macular edema (CME) and trace epiretinal membrane (ERM) on clinical examination and OCT imaging [Figure 1B]. There was a 40% gas fill in the eye at that time. Conservative management was recommended with face-down positioning for 10 days. The patient remained on topical prednisolone drops four times per day and his previously prescribed timolol and latanoprost. By postoperative month 2, the MH was found to have closed and has remained closed through the most recent follow-up, postoperative month 6 [Figure 1C]. Visual acuity at the time of MH closure was 20/60. At the most recent follow-up, the patient's vision in the left eye was 20/25.

### Case 2

**Figure 2:**
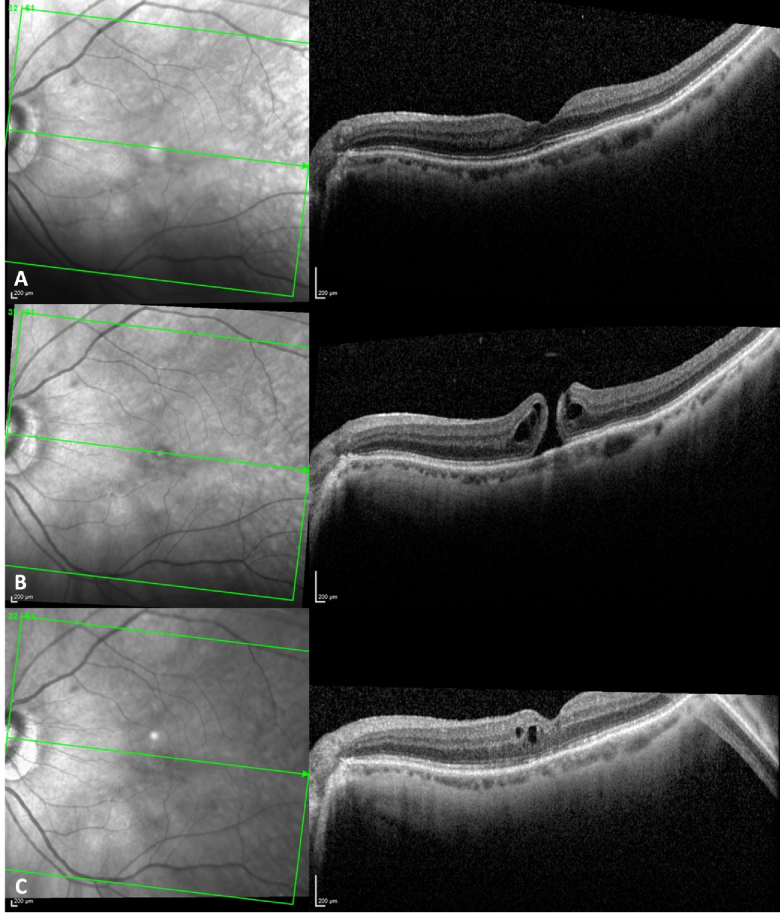
Sequential optical coherence tomography (OCT) demonstrating normal macula architecture following vitrectomy for patient 2 (A). At postoperative month 2, the patient demonstrated a MH (measuring 166 µm) (B). By postoperative month 4, the MH was found to have closed (C).

A 67-year-old female with a history of primary open-angle glaucoma in both eyes (prescribed timolol once per day in the morning and latanoprost nightly in both eyes), pseudophakia in both eyes, and RRD in the right eye (status-post scleral buckling and PPV in 2009) presented with several days of flashing lights and floaters in her left eye. On examination, the best-corrected visual acuity was 20/30 in the right eye and 20/20 in the left eye. Intraocular pressures were normal at 18 and 12 mmHg for the right and left eyes, respectively. Pupillary examination was without a relative afferent pupillary defect in either eye. On dilated fundus examination, a superior macula-sparing RRD was identified. The patient subsequently underwent scleral buckling, 25-gauge PPV, fluid–air exchange, endolaser, and 15% C3F8. Surgery was successful [Figure 2A]. On postoperative day 1, the patient's intraocular pressure was elevated to 24, and she was started on dorzolamide twice per day for pressure management in addition to the timolol and latanoprost the patient was already taking for glaucoma.

At postoperative month 2, the patient returned for follow-up. Her visual acuity was 20/50, and she was noted to have a small, full-thickness MH (measuring 166 µm minimum aperture diameter) with trace CME and ERM on clinical examination and OCT imaging [Figure 2B]. The decision was made to observe the MH with close follow-up. At the time of diagnosis, the patient was taking prednisolone drops four times per day and was instructed to taper off the drops over the course of 3 weeks. The patient remained on dorzolamide, timolol, and latanoprost. The full-thickness MH in the left eye was stable at postoperative month 3. However, by postoperative month 4, the MH was found to have closed spontaneously and has remained closed through the most recent follow-up at postoperative year 7 [Figure 2C]. The patient's vision at the time of MH closure and most recent follow-up was 20/40.

##  LITERATURE REVIEW

A search of PubMed, Medline, Science Direct, Web of Science, Cochrane Library, and Google Scholar for all years through November 2024 for articles written in English using the terms “spontaneous closure”, “macular hole”, and “vitrectomy” was performed. Additional studies were identified by reviewing article references and similar articles linked in PubMed. After removing duplicate reports, the articles were reviewed. Studies were included if they reported one or more cases of full-thickness, fovea-involving MHs that developed following vitrectomy surgery and were reported to have spontaneously closed. Cases were excluded if the patient had a history of a lamellar MH, full-thickness MH, VMT, trauma prior to vitrectomy, or if the MH was closed surgically. Cases were also excluded if data were only represented in aggregate and individual case characteristics were not identifiable in the text or tables.

Eighteen articles published between 2006 and 2024 were identified and reviewed.^[6,7,8,9,10,11,12,13,14,15,16,17,18,19,20,21,22,23]^ When available, the demographic and clinical data collected included patient age, sex, surgical history and indication, time to MH formation and spontaneous closure, MH size, presence of an ERM, posterior vitreous cortex remnants, CME, whether the MH recurred, time to MH recurrence, and length of follow-up after MH diagnosis. Data is summarized in Table 1.

A total of 34 eyes of 33 patients were identified, including the 2 patients we present here. The mean age at initial presentation was 57.6 years (range, 12-80 years). Most patients (28 eyes, 82.4%) had a history of vitrectomy for RRD. Of these 28 eyes, 13 (46.4%) had a history of macula-sparing RRD, 10 (35.7%) had a history of macula-involving RRD, and the status of the macula at the time of RRD repair was not specified for 5 (17.9%). Additionally, 6 of the 28 eyes (21.4%) that underwent RRD repair had PPV with concurrent scleral buckle (SB) placement. Other indications for PPV included vitreous hemorrhage (VH) in two eyes (7.1%). The underlying etiology for the VH in these cases was a retinal tear and a ruptured retinal arterial macroaneurysm, respectively. One patient underwent PPV for intraocular lens dislocation in both eyes. One patient underwent PPV with radial optic neurotomy for central retinal vein occlusion, and another patient underwent tractional retinal detachment repair with ILM peeling. The time from MH diagnosis to the most recent follow-up visit varied, with an average follow-up of 22.6 ± 28.8 months.

The time from PPV to MH diagnosis varied greatly, with an average time of 21.5 ± 31.7 months (median, 8 months; range, postoperative day 1 to 137 months). However, the time to spontaneous MH closure following diagnosis was relatively short, with a mean time to MH closure of 2.5 ± 3.2 months (median, 1 month). In total, 63.0% of eyes demonstrated MH closure within 1 month and 81.5% within 3 months.

Concurrent macular pathology at the time of MH formation was seen in most of these eyes. Among eyes with available data, 96.8% had concurrent macular pathology at the time of MH diagnosis. Moreover, 20 eyes (58.8%) had an ERM present at the time of MH development, 20 (58.8%) had CME present on OCT imaging, and 4 (11.8%) had posterior vitreous cortex remnants with associated VMT. All patients with residual vitreous leading to VMT had closure of MH following spontaneous release of the posterior vitreous and resolution of the VMT. This release was seen within 1 month postoperatively in all cases. Three eyes (8.8%) in the series did not have this clinical data documenting the presence or absence of concurrent macular pathology.

**Table 1:** Demographic and clinical characteristics of reported cases

**Study**	**Year**	**Patient number**	**Eye number**	**Age at presentation (years)**	**Sex**	**Surgical history**	**Surgical indication**	**Time to MH diagnosis following surgery (months)**	**Time to MH closure (months)**	**MH size (µm)**	**ERM present**	**Posterior vitreous cortex remnants present**	**CME present**	**Recurrent MH development**	**Time to MH recurrence (months)**	**Follow up from MH diagnosis (months)**
Lo & Hubbard^[6]^	2006	1	1	69	F	PPV	VH (RAMA)	83	4	N/A	No	No	Yes	Yes	19	23
Shukla et al^[7]^	2006	2	2	45	F	PPV/RON	CRVO	0.03	1	N/A	No	No	Yes	No	–	11
Tsilimbaris et al^[8]^	2007	3	3	53	F	(1) SB, (2) PPV, (3) PPV	Recurrent MI-RRD	0.33	9	N/A	N/A	N/A	N/A	No	–	N/A
Tsilimbaris et al^[8]^	2007	4	4	60	F	PPV	RRD	2	9	N/A	No	No	Yes	No	–	N/A
Tsilimbaris et al^[8]^	2007	5	5	55	M	PPV/SB	RRD	24	12	N/A	N/A	N/A	N/A	No	–	N/A
Fabian et al^[9]^	2012	6	6	53	F	PPV	MI-RRD	48	1	N/A	Yes	No	Yes	No	–	6
Kim & Park^[10]^	2015	7	7	58	M	PPV	MI-RRD	0.5	0.5	N/A	No	Yes	Yes	No	–	0.5
Khurana et al^[11]^	2017	8	8	67	F	PPV/SB	MI-RRD	4	1	74	Yes	No	Yes	No	–	16
Khurana et al^[11]^	2017	9	9	58	M	PPV	MS-RRD	21	1	222	Yes	No	Yes	No	–	9
Sridhar et al^[12]^	2017	10	10	59	M	PPV	MS-RRD	2	1	N/A	No	No	Yes	Yes	2	5
Zhang et al^[13]^	2017	11	11	47	M	PPV/SB	MS-RRD	36	2	189	Yes	No	Yes	No	–	120
Zhang et al^[13]^	2017	12	12	61	F	PPV/ILM peel	TRD	7	0.5	48	Yes	No	Yes	No	–	12
Mori et al^[14]^	2018	13	13	66	M	PPV	IOL dislocation	10	1	N/A	Yes	No	Yes	Yes	52	78
Mori et al^[14]^	2018	13	14	80	M	PPV	IOL dislocation	75	3	N/A	No	No	Yes	Yes	21	24
Mori et al^[14]^	2018	14	15	N/A	M	PPV	RRD	33	3	N/A	No	No	Yes	Yes	4	7
Ranjan et al^[15]^	2018	15	16	57	M	PPV/SO	MI-RRD	0.03	0.5	N/A	No	No	No	No	–	3
Nam et al^[16]^	2020	16	17	66	F	PPV	MS-RRD	8	0.5	N/A	Yes	No	Yes	No	–	1
Ozdemir & Ozdek^[17]^	2020	17	18	45	F	(1) PPV/SO, (2) SOR	MI-RRD	18	2	N/A	Yes	No	N/A	Yes	10	12
Massamba et al^[18]^	2020	18	19	49	M	PPV/SB	RRD	N/A	1	N/A	Yes	No	Yes	Yes	4	N/A
Siddiqui et al^[19]^	2021	19	20	N/A	N/A	PPV	RRD	0.75	8	N/A	N/A	N/A	N/A	No	–	20.25
Uemura et al^[20]^	2021	20	21	54	F	PPV	MI-RRD	12	N/A	183	Yes	No	N/A	No	–	21
Uemura et al^[20]^	2021	21	22	56	M	PPV	MS-RRD	23	N/A	109	Yes	No	N/A	No	–	11
Uemura et al^[20]^	2021	22	23	55	M	PPV	MS-RRD	1	N/A	211	Yes	No	N/A	Yes	N/A	68
Uemura et al^[20]^	2021	23	24	51	F	PPV	MS-RRD	36	N/A	147	Yes	No	N/A	Yes	N/A	16
Uemura et al^[20]^	2021	24	25	68	M	PPV	MS-RRD	38	N/A	88	Yes	No	N/A	Yes	N/A	38
Uemura et al^[20]^	2021	25	26	58	M	PPV	MS-RRD	80	N/A	135	Yes	No	N/A	Yes	N/A	48
Uemura et al^[20]^	2021	26	27	70	M	PPV	MS-RRD	137	N/A	111	Yes	No	N/A	Yes	N/A	20
Komi et al^[21]^	2023	27	28	64	F	PPV	VH (RT)	8	0.5	N/A	Yes	No	Yes	Yes	13	10
Pellegrini et al^[22]^	2020	28	29	69	M	PPV	MS-RRD	0.75	0.75	N/A	Yes	No	Yes	No	–	4.5
Xu et al^[23]^	2024	29	30	12	F	PPV	MI-RRD	0.25	0.5	N/A	No	Yes	Yes	No	–	0.75
Xu et al^[23]^	2024	30	31	67	M	PPV	MI-RRD	0.25	0.75	558	No	Yes	No	No	–	3
Xu et al^[23]^	2024	31	32	31	F	PPV	MI-RRD	0.5	1	897	No	Yes	No	No	–	2.5
Bonnell et al^[26]^*	2022	32	33	72	M	(1) PPV, (2) PPV/SB	MS-RRD	1	1	61	Yes	No	Yes	No	–	5
Bonnell et al^[26]^*	2022	33	34	67	F	PPV	MS-RRD	2	2	166	Yes	No	Yes	No	–	82
*This unpublished case series includes all patients at the University of Washington. CME, cystoid macular edema; CRVO, central retinal vein occlusion; ERM, epiretinal membrane; F, female; ILM, internal limiting membrane; IOL, intraocular lens; M, male; MH, macular hole; MI, macula-involving; MS, macula-sparing; N/A, not available; PPV, pars plana vitrectomy; RAMA, retinal artery microaneurysm; RON, radial optic neurotomy; RRD, rhegmatogenous retinal detachment; RT, retinal tear; SB, scleral buckle; SO, silicone oil; SOR, silicone oil removal; TRD, tractional retinal detachment; VH, vitreous hemorrhage.																

Thirteen eyes (38.2%) developed recurrent MH following closure. Of them, 9 had ERM, with or without CME, and 4 had CME alone. Data are available for the time to MH recurrence for eight of these eyes. The mean time to MH recurrence in these cases was 15.6 ± 16.3 months (range, 2 to 52 months).

Overall, the MHs in this group were small, with a mean size of 213.3 ± 224.8 µm. Of note, MHs in the setting of VMT from residual vitreous were larger in all cases as compared to the other MHs, with a mean MH size of 727.5 ± 239.7 µm vs. 134.2 ± 57.7 µm, respectively (*P* < 0.0001).

##  DISCUSSION

The development of MHs following PPV is rare, with an estimated incidence of < 1%.^[4,24]^ MHs have been observed as early as the first postoperative day to as late as 11 years after PPV. In these cases, a surgeon may consider returning to the operating room for surgical repair or observing for spontaneous closure. In one study, the incidence of spontaneous MH closure in vitrectomized eyes for RRD repair was about 15%.^[5]^ While observation may be appropriate in some instances, there is limited knowledge regarding the characteristics of spontaneous MH closure in eyes following PPV. This report presents two cases of MH formation and closure following PPV and reviews the literature, examining the demographic and clinical characteristics of these eyes. This study presents the most comprehensive review of this topic to date.

Some have previously considered the etiology of MH development following PPV.^[5,13]^ The three primary hypotheses for MH formation that have been described include (1) anterior-posterior traction at the macula either at the time of surgery or from residual posterior vitreous remnants left after surgery; (2) ERM proliferation with tangential retinal traction; and (3) CME leading to disruption of the foveal architecture. The findings of this study suggest that all three of these mechanisms may be at play. In this report, among eyes with available data, all but one eye (96.8%) had at least one of these features present at the time of MH development, with ERM and CME being the most common conditions, each seen in 58.8% of the cohort.

The average size of MHs that closed spontaneously was small (213.3 µm). As other studies suggest, spontaneous closure may be more common among small MHs. In a retrospective study by Starr et al, MHs with spontaneous closure were smaller, on average, compared to those that did not close on their own (161.8 vs. 588.7 µm).^[5]^ Therefore, a period of observation for small to medium MHs ( < 400 µm) may be considered to allow for spontaneous closure.

Eyes that develop MHs in the setting of VMT from posterior vitreous remnants may represent a unique subset within these types of post-vitrectomized MH. These holes were larger in all cases, with an average size of 727.5 µm compared to 134.2 µm in eyes without residual vitreous (*P* = 0.0001). MH closure was seen in all eyes following spontaneous VMT release. This release was seen by postoperative month 1 in all cases. Therefore, observing even large MHs ( > 400 µm) may be reasonable if the etiology of MH development is VMT from residual vitreous. Whereas a large hole without this finding may prompt earlier surgery.

Overall, the time from MH diagnosis to MH closure was short. The median time to MH closure was 1 month, ranging from 2 weeks to 1 year. It is important to note that this number may be an underestimation of the average time to closure, given that a patient may elect for surgical repair if an MH does not close within a relatively short observation period. Nevertheless, 63.0% of all cases in this review demonstrated MH closure within 1 month of diagnosis, and 81.5% of MHs closed within 3 months of diagnosis. When a surgeon is considering a reasonable time frame for observation, 1 to 3 months following diagnosis may be appropriate since the vast majority of reported MHs closed spontaneously within this period.

Recurrent MH development after spontaneous closure was not uncommon, seen in 38.2% of cases. Although data in this small cohort are limited, there does not appear to be a relationship between the presence of ERM or CME and MH recurrence. The time to MH recurrence ranged from 2 to 52 months, with an average of 15.6 months. It is important to note that the average follow-up in eyes without recurrence was short at 18.3 months, with a median follow-up time of 7.5 months. With longer follow-up, the measured rate of MH recurrence may be higher. While some of these eyes went on to have repeated spontaneous closure, some eyes required additional surgery to achieve closure. Surgeons and patients should be aware that over one-third of holes may reopen after spontaneous closure, and continued close observation of these eyes is prudent.

Medical therapies for MH closure have been explored in recent years. The rationale for medical therapy is based on the “hydration theory” of MH development and closure, which postulates that edema at the fovea disrupts the retinal architecture, propagating MH formation.^[25]^ Medical therapies include local steroids, nonsteroidal anti-inflammatory medications, and carbonic anhydrase inhibitors. There is a growing amount of evidence to suggest that these medications may help facilitate MH closure in cases with CME by reducing the cystoid fluid, restoring the normal retinal architecture at the edges of the MH, and allowing the edges of the hole to come together and close. ${}^{[}$ 26-28 ${}^{]}$ Many patients in this cohort presented within the immediate postoperative period, where topical steroids and other agents may have been used. In the two cases reported here, one patient remained on a topical carbonic anhydrase inhibitor and the other a topical steroid. We must consider that these agents played a role in the closure of these MH.

The role of gas tamponade must also be considered in the cases of MH closure. For example, at the time of MH formation for Case 1, the patient had 40% C ${}_{3}$ F ${}_{8}$ gas fill. The patient was instructed to position facedown for 10 days following identification of the MH. The MH was noted to have closed by postoperative month 2. Gas tamponade is understood to play an important role in MH closure. It is believed that gas tamponade helps to promote MH closure by providing physical support to the retina and providing a scaffold for glial cell proliferation.^[29]^ For the cases identified by the literature review, it is not known if there was gas tamponade present at the time of MH closure and if positioning was utilized in these cases. However, a surgeon may consider instructing a patient to position facedown if gas is present in the eye at the time of MH identification.

There are several limitations of this report. Firstly, although this is the largest review of its kind, it describes only a small number of patients. By the nature of this report, there is no control group for comparison. Patients in this study have been examined retrospectively, leading to bias. Potential areas for bias have been explained in this discussion, including a likely underestimation of time to MH closure and rates of MH recurrence. As with any literature review, our review may not have captured all published studies and reports.

In summary, the development of an MH in a vitrectomized eye is rare. MH development has been seen as early as postoperative day 1 following PPV to as late as 11 years after surgery. When observed in other series, the chance that an MH will close may be estimated to be about 15%.^[5]^ A small or medium MH ( < 400 µm) may be more likely to close spontaneously. MHs that form in the setting of VMT from residual posterior vitreous cortex remnants are larger ( > 400 µm) and have been found to close after spontaneous VMT release, often within the first month following surgery. Overall, among eyes with spontaneous closure, about two-thirds of MHs in the literature closed spontaneously within the first month following diagnosis, and four in five holes closed within 3 months. Over one-third of MHs that close spontaneously reopen later, some requiring additional surgery to achieve closure.

##  Financial Support and Sponsorship

None.

##  Conflicts of Interest

None.
